# Comparative analysis of fecal microbial community diversity between Bactrian camels and Mongolian cattle based on 16S rRNA gene sequencing

**DOI:** 10.3389/fmicb.2026.1898199

**Published:** 2026-07-24

**Authors:** Xueqin Ma, Wenyi Ren, Shuangming Yang, Miao Liu, Weihao Kong, Xiaofeng Xu, Lili Zhang

**Affiliations:** School of College of Animal Science and Technology, Ningxia University, Yinchuan, China

**Keywords:** 16S rRNA, arid desert grazing, Bactrian camel, gut microbiota, Mongolian cattle

## Abstract

**Introduction:**

The Alxa region is a typical arid and semi-arid ecosystem in northwestern China, where Bactrian camels and Mongolian cattle coexist under natural grazing conditions but differ markedly in feeding behavior and environmental tolerance. These differences may be reflected in host-associated gut microbial communities. This study compared the fecal microbiota of the two species to clarify microbial features associated with adaptation to the local desert grassland environment.

**Methods:**

Fecal samples were collected from 12 healthy adult Bactrian camels and 12 healthy adult Mongolian cattle grazing on spring pasture in the Alxa region. Total microbial DNA was extracted from fecal samples, and the hypervariable region of the bacterial 16S rRNA gene was amplified and sequenced using the Illumina platform. Community composition, alpha and beta diversity, differential taxa and microbial co-occurrence networks were then analyzed.

**Results:**

The Shannon index was significantly higher in Bactrian camels than in Mongolian cattle (*P* = 0.049), whereas the Simpson index showed an opppsite trend (*P* = 0.013). PCoA and NMDS analyses further showed significant separation between the fecal microbial communities of the two host species (*P* = 0.001). At the phylum level, *Bacillota* and *Bacteroidota* dominated fecal samples from both species. At the genus level, the dominant taxa in Bactrian camels included *Rikenellaceae_RC9_gut_group, norank_f_UCG-010, Christensenellaceae_R-7_group, UCG-005, Treponema and Bacteroides*, whereas Mongolian cattle were dominated mainly by *UCG-005, Rikenellaceae_RC9_gut_group* and *norank_f_UCG-010*. Co-occurrence network analysis showed that the camel microbial network had higher edge number, graph density and average degree, but lower network diameter, clustering coefficient and modularity, than the cattle network. The core nodes were *UCG-005, Rikenellaceae_RC9_gut_group* and *Succinivibrio* in Bactrian camels, and *Rikenellaceae_RC9_gut_group, Bacteroides RF16 group* and *Prevotella* in Mongolian cattle.

**Discussion:**

These findings provide preliminary microbial evidence that Bactrian camels and Mongolian cattle maintain their survival by shaping distinct gut microbial community structures and functional interaction patterns.

## 1 Introduction

Alxa League in Inner Mongolia is located in the arid zone of northwestern China, where desert and semi-desert landscapes dominate the ecosystem. Plants in this region have sparse leaves, low stature and low biomass, and they contain large amounts of cellulose and lignin that are difficult for animals to digest and absorb, especially at later growth stages when lignification becomes more pronounced. This distinctive environment has shaped a characteristic regional biodiversity, including drought-tolerant and roughage-adapted livestock such as Mongolian cattle and Bactrian camels. Mongolian cattle are an important species in grassland animal husbandry and mainly feed on low-growing herbs and shrubs, such as *Achnatherum splendens, Agropyron desertorum, Leymus secalinus* and tender branches and leaves of *Haloxylon ammodendron*. By contrast, Bactrian camels are key species in desert ecosystems and have a broader dietary range, allowing them to consume coarse shrubs, salt-rich plants and plants with certain toxic properties, such as *Agriophyllum squarrosum, Tamarix chinensis, Stellera chamaejasme* and *Amygdalus mongolica* (Zhang et al., 2020). These differences in survival strategy and feeding habit may profoundly affect the structure and function of their digestive tract microbial communities.

Taxonomically, camels and cattle are closely related and both are generally classified among ruminant suborder. However, the system of Bactrian camels is significantly different from that of other typical ruminants. The digestive system of camels differs significantly from that of other typical ruminants. Their stomach consists only of the rumen, reticulum and omasum, lacking the omasum, and the fore-stomach has specialized glandular areas. The gut microbiota of ruminants is a complex microbial community that contributes directly to host nutrient digestion and absorption, energy metabolism, immune regulation and enzymatic metabolism, mainly by promoting the fermentation of otherwise indigestible plant material (Davis et al., 2022). It supplies approximately 70% of host energy requirements and 60–85% of amino acids entering the small intestine (Keum et al., 2024). Differences in the microbial composition of the digestive tracts of different ruminant animals are closely related to the host's genetic background, diet, age and environmental adaptation (Zhang et al., 2021). Carroll et al. (2025) compared the microbial flora of calves before and after weaning and found that the relative abundances of *Pseudomonadota* and *Bacteroidota* decreased after weaning, whereas *Bacillota* increased, these changes in phyla suggest that the dietary structure transformation may affect the rumen and intestinal microbial communities. Li et al. (2020) compared the microbial flora in dairy cow feces in spring and summer and found that during summer heat stress, the Chao1 and ACE indices of microbial abundance showed a downward trend, and the Shannon and Simpson indices were significantly lower than those in spring, indicating that environmental changes have a significant inhibitory effect on the microbial community. Furthermore, environmental factors may also have a profound impact on the gut microbiota. Reyes et al. (2026) conducted a study on the beef cattle microbiota in six subregions of Colombia and found that although there was a core microbiota group in this region, there were still significant differences in the community composition among different subregions. However, after excluding factors such as feed type, season and altitude, environmental factors can still independently explain approximately 9 to 10% of the community changes. This result suggests that when the environmental background is strictly uniform, the host's own genetic background will become the key driving force shaping the microbial diversity and co-occurrence network. Reyes et al. (2025)'s study on Japanese black cattle indicates that the farm location explains 27.1 to 38.9% of the β-diversity variation of the rumen microbiome, while fertility explains only 3.2 to 4.0%. This result suggests that when comparing the intestinal microbiomes of different species, the consistency of the environmental background must be strictly controlled; otherwise, geographical environmental differences may completely overshadow the effect of the host species.

In this study, samples were collected from both Bactrian camels and Mongolian cattle under exactly the same pasture conditions, aiming to minimize environmental interference and focus on the shaping effect of host species on the microbial community. The composition and diversity differences of the fecal microbial communities were analyzed using high-throughput sequencing technology of the 16S rRNA gene, providing a theoretical basis for studying the adaptability of animals in extreme environments and the mechanism of animal-microbe interaction. Cattle under exactly the same pasture conditions, aiming to minimize environmental interference and focus on the shaping effect of host species on the microbial community. The composition and diversity differences of the fecal microbial communities were analyzed using high-throughput sequencing technology of the 16S rRNA gene, providing a theoretical basis for studying the adaptability of animals in extreme environments and the mechanism of animal-microbe interaction.

## 2 Materials and methods

### 2.1 Experimental design

The study was approved by the Animal Ethics Committee of Ningxia University (NXU-2026–129). The experimental animals were 12 Mongolian cattle and 12 Bactrian camels from Alxa League, Inner Mongolia. Sampling was conducted in an area located between 97°10′ E to 106°53′ E and 37°24′ N to 42°47′ N, situated in the western part of the Inner Mongolia Plateau, where annual precipitation is relatively low. The local vegetation is pre-dominantly composed of xerophytic and ultra-xerophytic shrubs and semi-shrubs. All animals were raised under natural grazing conditions throughout the year, were healthy and disease-free, and had not used probiotics or antibiotics within 2 months prior to sampling. Before morning feeding, after separate preservation of Mongolian cattle and Bactrian camels, the sampling personnel wore sterile gloves and inserted a rectal scraper through the anus into the rectum about 10 to 15 cm to scrape the fresh fecal contents in the middle section. For each individual, approximately 5 to 10 grams of fecal samples are collected. After quickly removing any impurities such as hair and grass debris mixed in, they are immediately aliquot into sterile cryotubes, with each tube containing about 2 to 3 grams. And within 3 min after collection, it was rapidly frozen in liquid nitrogen, and then transferred to a −80 °C refrigerator for storage, which was used for subsequent DNA extraction and analysis.

### 2.2 Microbial DNA extraction and 16S rRNA gene sequencing

Total microbial DNA was extracted using the E.Z.N.A. soil DNA Kit. DNA quality was assessed by 1% agarose gel electrophoresis, and DNA concentration and purity were quantified using a NanoDrop 2000 spectrophotometer. Qualified DNA was used as the template for PCR amplification with barcode-tagged specific primers. The V3-V4 hypervariable region of the bacterial 16S rRNA gene was amplified using the universal primer pair 338F 5′-ACTCCTACGGGAGGCAGCAG3′ and 806R 5′-GGACTACNVGGGTWTCTAAT3′. The PCR protocol consisted of initial denaturation at 95 °C for 3 min, followed by 27 cycles of denaturation at 95 °C for 30 s, annealing at 55 °C for 30 s and extension at 72 °C for 30 s, with a final extension at 72 °C for 10 min. PCR products were recovered and purified from 2% agarose gels, followed by quality control and library construction. Paired-end sequencing was performed on the Illumina NextSeq 2000 platform (Illumina, USA).

### 2.3 Bioinformatics and statistical analysis

Raw paired-end sequences were first quality-controlled using fastp (version 0.23.1) to trim low-quality bases and filter reads. Subsequently, the QIIME2 (version 2022.2) dada2 denoise-paired plugin was employed for sequence processing, which performed quality filtering, error modeling, denoising, and the merging of paired-end reads based on their overlap relationships in a single integrated workflow, following the DADA2 standard pipeline. The resulting amplicon sequence variants (ASVs) were then obtained. Taxonomic assignment was performed by comparing the ASV representative sequences against the SILVA 138.2 16S rRNA database (99% similarity) using the classify-sklearn naive Bayes classifier within QIIME2 at different taxonomic levels.

Prior to diversity analysis, all samples were rarefied to an even sequencing depth of 29109 reads per sample based on the minimum library size to normalize sampling effort. Alpha diversity indices were calculated using Mothur (version 1.48.0). PCoA based on Bray-Curtis distance, combined with ANOSIM was used to evaluate the overall difference of bacterial communities among different groups. A Venn online analysis tool (https://bioinfogp.cnb.csic.es/tools/venny/, version 2.4.3) was used to identify unique and shared ASVs among sample groups, and community heatmap analysis based on the relative abundance of top genera was used to display species distribution patterns. Wilcoxon rank-sum tests in R were performed to evaluate significant differences in the relative abundance of microbial taxa between the two groups, and LEfSe (Linear Discriminant Analysis Effect Size, version 1.1.0) analysis was further used to screen taxa with significant between-group effects, with an LDA score threshold of 2.0. Microbial co-occurrence networks were constructed based on Spearman correlation coefficients (|ρ| > 0.6, *P* < 0.05) calculated in R, and visualized using Gephi (version 0.11.1). Quantitative data are expressed as mean ± standard deviation. *P* < 0.05 was considered statistically significant between the two groups.

## 3 Results

### 3.1 Assessment of 16S rRNA gene sequencing

**Figure 1:**
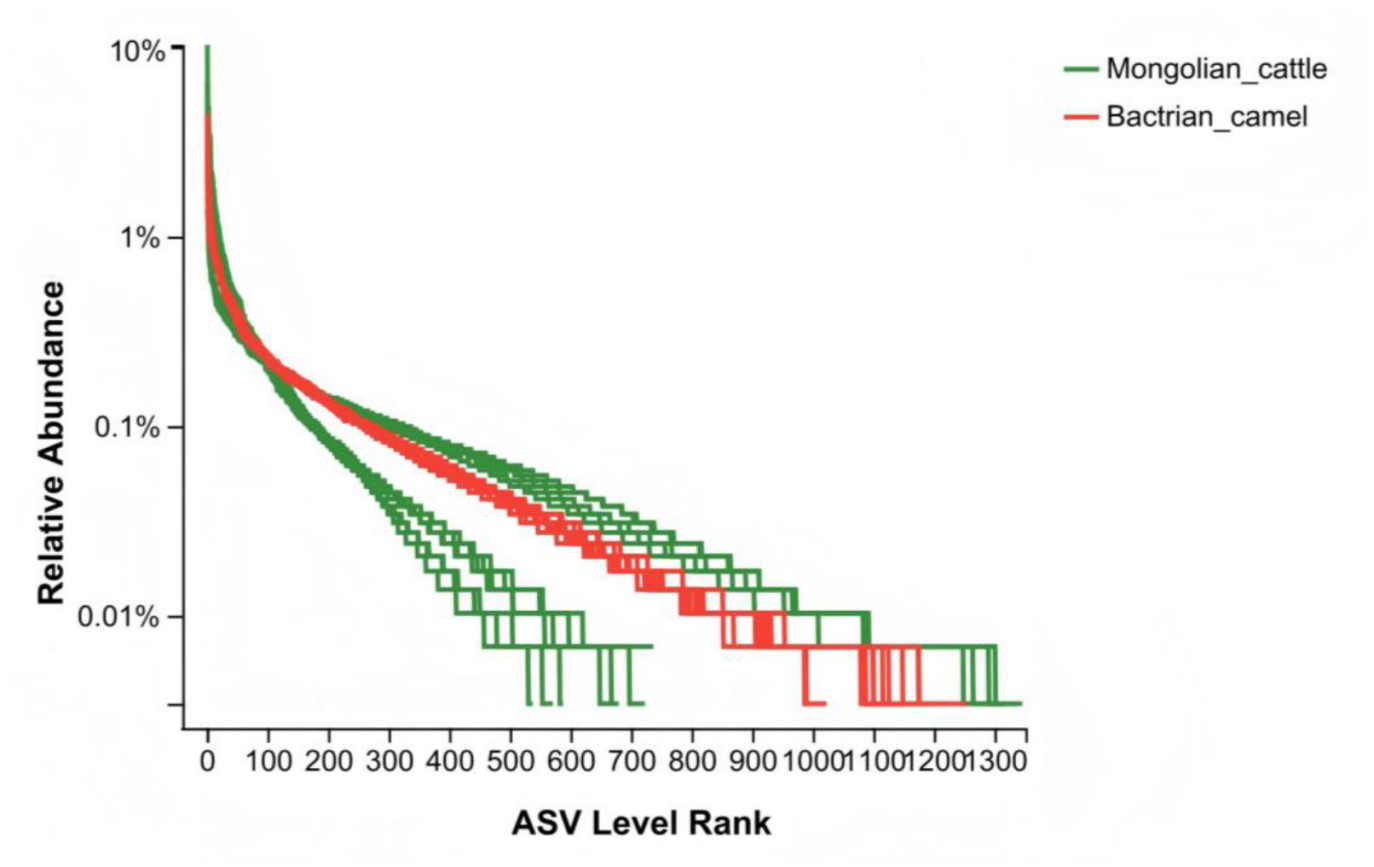
Clustering curves of microbial levels in the feces of Mongolian cattle and Bactrian camels.

A total of 761,024 high-quality controlled sequences were obtained from 24 animal fecal samples by bacterial 16S rRNA sequencing, with an average length of 414 bp. As shown in Figure 1, the rank-abundance curves of both groups were broad and eventually tended to plateau, indicating that the samples were compositionally even and rich. Bactrian camel samples showed a relatively concentrated distribution, whereas some variation was observed among Mongolian cattle samples. In alpha diversity analysis, the Shannon index is commonly used to evaluate microbial diversity within samples.

### 3.2 Alpha diversity analysis

ACE, Chao 1, Shannon and Simpson indices were used to reflect microbial community richness and diversity. As shown in Table 1, the ACE and Chao 1 indices were higher in Bactrian camel samples than in Mongolian cattle samples, although the differences were not significant (*P* > 0.05). The Shannon index was significantly higher in Bactrian camel samples (*P* = 0.049), whereas the Simpson index showed an opppsite trend (*P* = 0.013). These results indicate differences in fecal microbial richness and diversity between the two animal groups, with Bactrian camel samples showing greater microbial richness and higher bacterial diversity. Coverage exceeded 99% in all samples, indicating that the sequencing depth met the requirements for detecting microbial community information in fecal samples.

**Table 1:** Comparison of alpha diversity in fecal microbiota between Mongolian cattle and Bactrian camels.

Items	Bactrian camel	Mongolian cattle	*P-*value
ACE	1,148.067 ± 3.16	931.653 ± 41.73	0.052
Chao 1	1,136.206 ± 8.68	9243 ± 36.75	0.052
Shannon	6.090 ± 0.06	5.690 ± 0.65	0.049
Simpson	0.00480 ± 0.0007	0.00960 ± 0.006	0.013
Coverage	0.99860 ± 0.0006	0.99820 ± 0.0007	0.157

### 3.3 Beta diversity analysis

**Figure 2:**
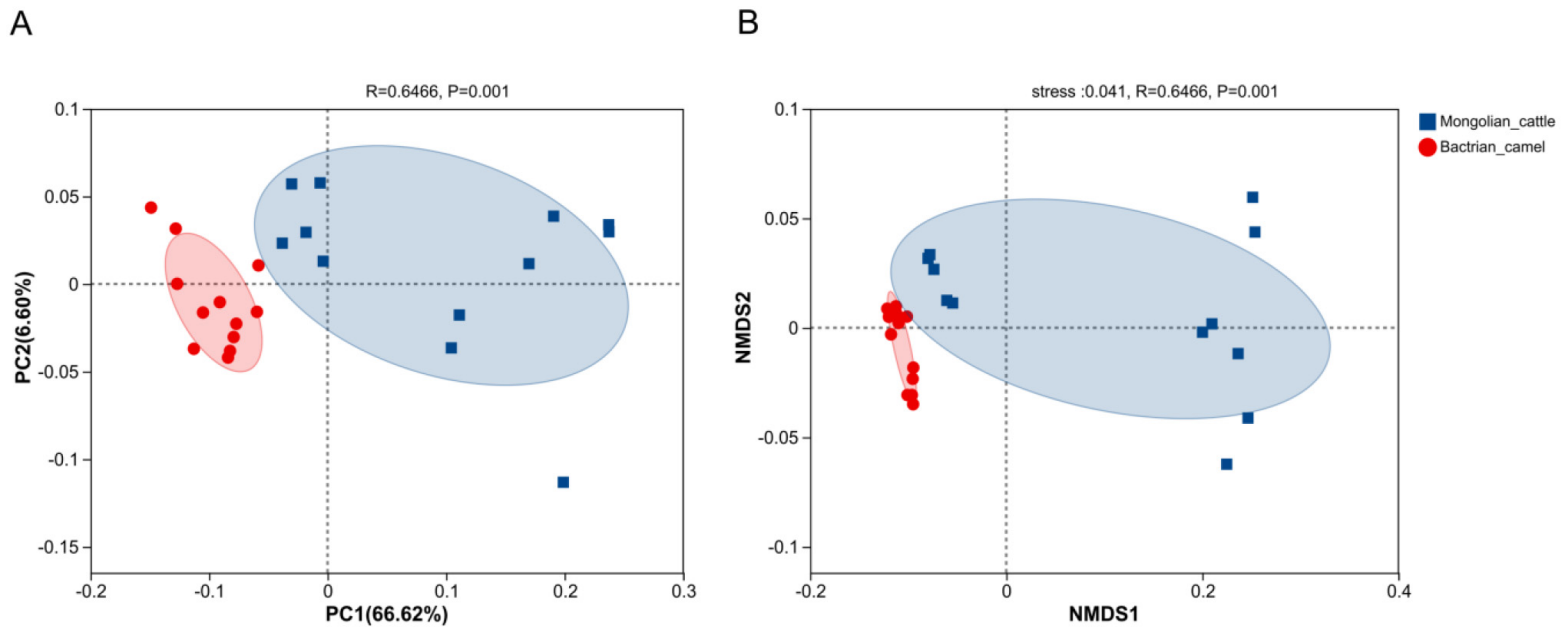
PCoA and NMDS analyses of fecal bacterial community structure in Mongolian cattle and Bactrian camels. **(A)** PCoA analysis of community structure. **(B)** NMDS analysis of community structure.

PCoA and NMDS analyses were performed based on Bray-Curtis distances. As shown in Figure 2A, principal components PC1 and PC2 explained 66.62 and 6.60% of the total variation, respectively. The microbial communities of Mongolian cattle and Bactrian camels were significantly separated (*P* = 0.001), indicating clear differences in gut microbial community structure between the two species. NMDS analysis (Figure 2B) further confirmed the PCoA results, with sample clustering showing host specificity.

### 3.4 Comparative analysis of fecal microbial community structure

#### 3.4.1 Relative abundance of fecal microbial taxa

**Figure 3:**
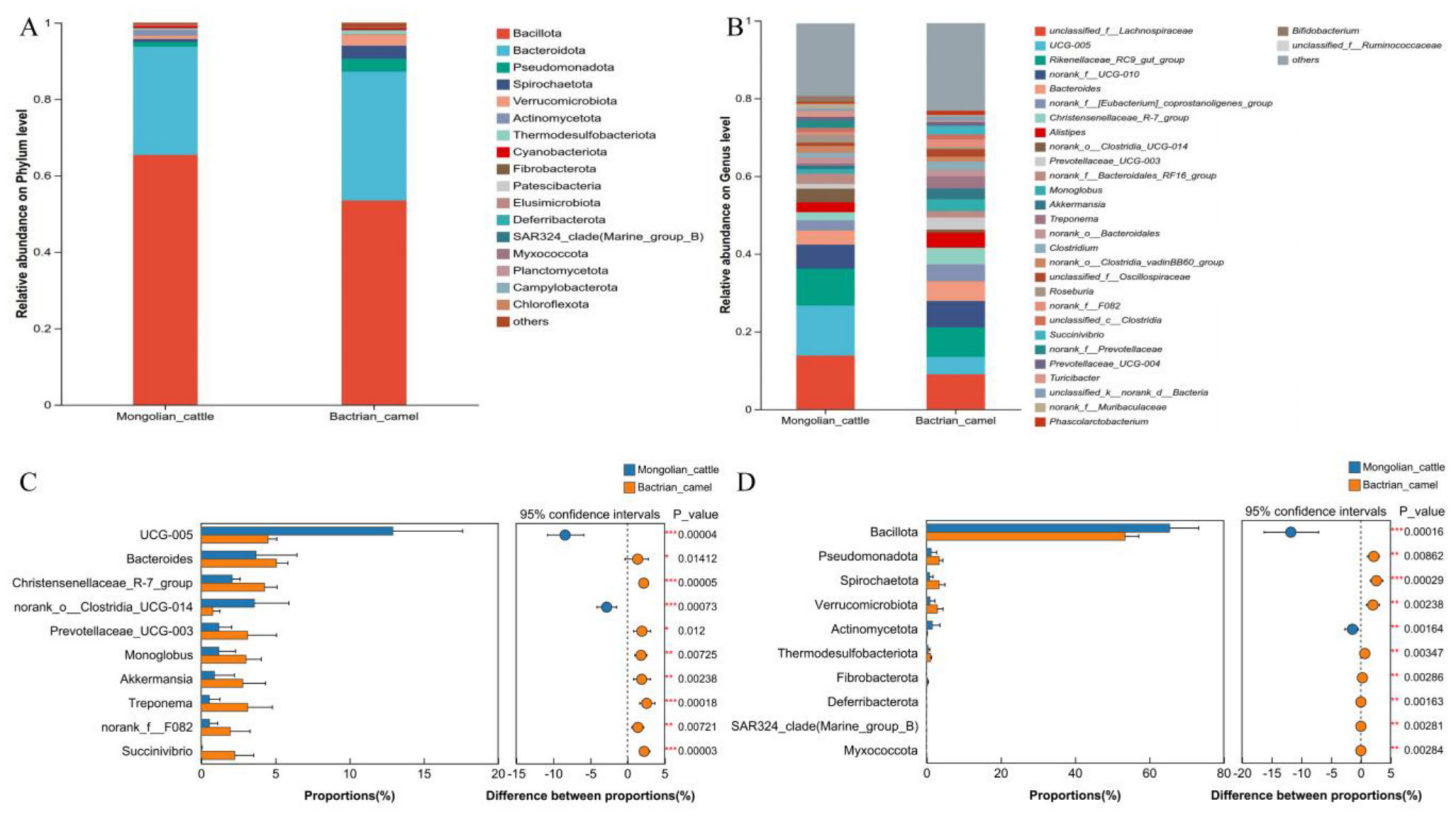
Comparative analysis of fecal microbial community structure in Mongolian cattle and Bactrian camels. **(A)** Phylum-level distribution of fecal microbial communities. **(B)** Genus-level distribution of fecal microbiota. **(C)** Wilcoxon rank sum test at the phylum level. **(D)** Wilcoxon rank sum test at the genus level.

At the phylum level (Figure 3A), the dominant phyla in Mongolian cattle were Bacillota (65.41%) and Bacteroidota (28.27%), followed by Pseudomonadota (1.24%), Spirochaetota (0.78%) and Verrucomicrobiota (0.90%). In Bactrian camels, the dominant phyla were also *Bacillota* (53.42%) and Bacteroidota (33.77%), followed by *Pseudomonadota* (3.40%), Spirochaetota (3.37%) and Verrucomicrobiota (2.90%).

At the genus level (Figure 3B), Mongolian cattle were mainly dominated by *UCG-005* (12.91%), *Rikenellaceae_RC9_gut_group* (9.46%), *norank_f_UCG-010* (6.16%), *norank_o_Clostridia_UCG-014* (3.58%)„ *Bacteroides* (3.70%). Bactrian camels were mainly dominated by *Rikenellaceae_RC9_gut_group* (7.63%), *norank_f_UCG-010* (6.82%), *Christensenellaceae_R-7_group* (4.27%), *UCG-005* (4.51%), *Treponema* (3.13%), *Bacteroides* (5.07%) and *Alistipes* (3.89%).

#### 3.4.2 Differential analysis of fecal microbiota at the phylum and genus levels

At the phylum level (Figure 3C), *Bacillota* and *Actinobacteriota* were significantly more abundant in Mongolian cattle samples than in Bactrian camel samples (*P* < 0.01), whereas *Pseudomonadota, Spirochaetota* and *Verrucomicrobiota* were significantly less abundant (*P* < 0.01).

At the genus level (Figure 3D), *UCG-005* and *norank_o_Clostridia_UCG-014* were significantly more abundant in Mongolian cattle samples than in Bactrian camel samples (*P* < 0.001), whereas *Christensenellaceae_R-7_group* and *Treponema* were significantly less abundant (*P* < 0.001).

### 3.5 LEfSe analysis

**Figure 4:**
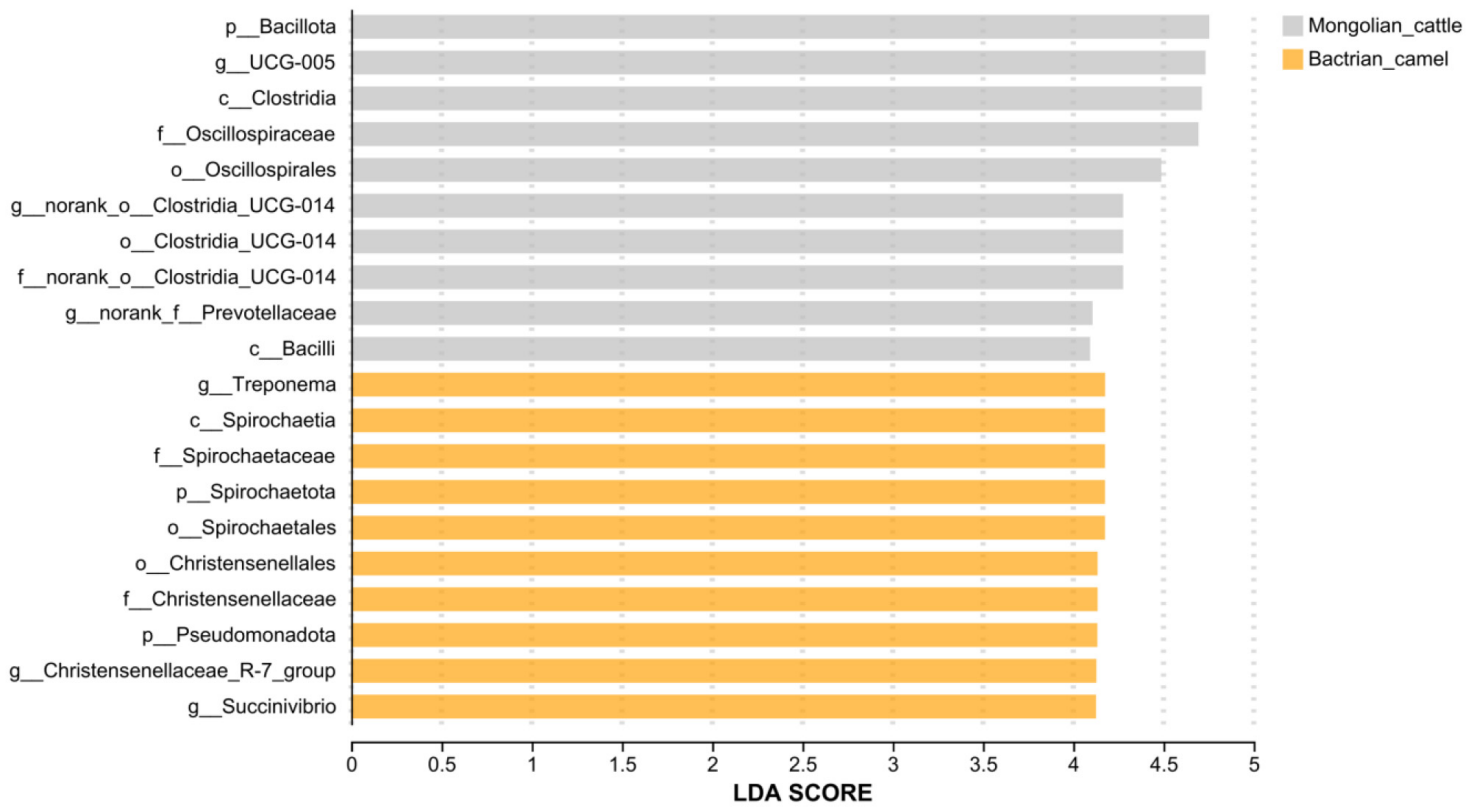
LEfSe analysis of fecal microorganisms in Mongolian cattle and Bactrian camels. The LDA score histogram evaluates significant differences in bacterial classification between Mongolian cattle and Bactrian camels. The y-axis indicates taxa with significant between-group differences, and the x-axis indicates the logarithmic LDA score for each taxon.

LEfSe analysis was performed on fecal samples from Bactrian camels and Mongolian cattle. The LDA results (Figure 4) showed that, at the genus level, each group contained 10 differentially abundant genera with an LDA score threshold greater than 4. In Mongolian cattle, characteristic changes were observed in one phylum, two classes, four orders, two families and one genus. The significantly altered phylum was *Bacillota*, and the significantly altered genus was *UCG-005*. In the Bactrian camel group, characteristic changes were observed in two phyla, one class, two orders, two families and three genera. The significantly altered phyla were *Spirochaetota* and *Pseudomonadota*, and the significantly altered genera were *Treponema, Christensenellaceae_R-7_group* and *Succinivibrio*.

### 3.6 Co-occurrence network analysis

**Figure 5:**
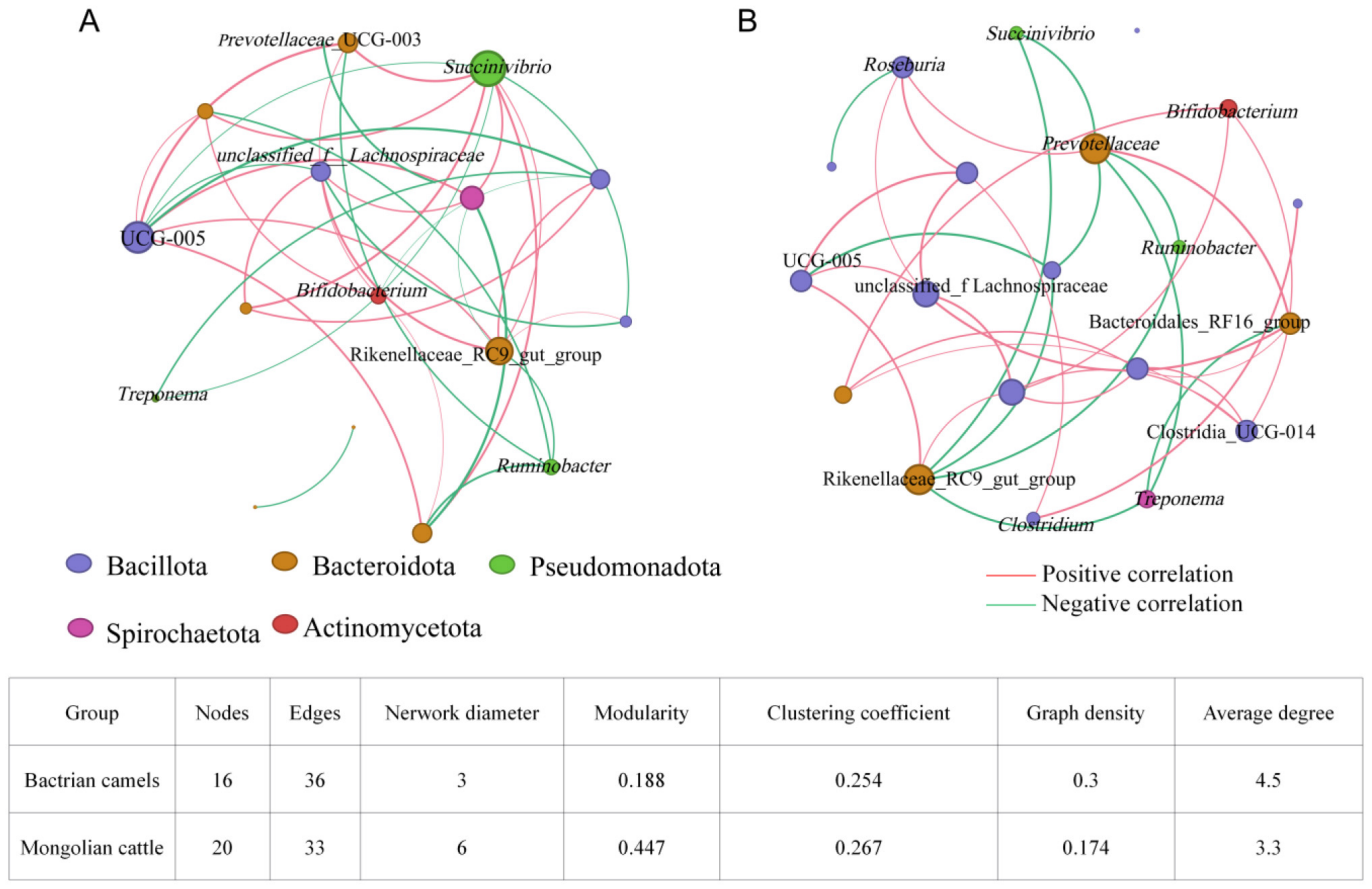
Gut microbial co-occurrence networks of Bactrian camels and Mongolian cattle. Each node in the co-occurrence network represents a single genus and is colored by phylum. **(A)** Gut microbial co-occurrence networks for Bactrian camels. **(B)** Gut microbial co-occurrence networks for Mongolian cattle. Edges between nodes indicate Spearman correlation coefficients |ρ| > 0.6 and *P* < 0.05; pink edges indicate positive associations, and green edges indicate negative associations. The table below shows the topological properties of the co-occurrence network.

By constructing gut microbial co-occurrence networks for Bactrian camels and Mongolian cattle, 16 and 20 network nodes were identified in Bactrian camels (Figure 5A) and Mongolian cattle (Figure 5B), respectively. Most nodes in both networks belonged to Bacillota and Bacteroidota. The core network nodes in Bactrian camels were *UCG-005, Rikenellaceae_RC9_gut_group* and *Succinivibrio*, whereas those in Mongolian cattle were *Rikenellaceae_RC9_gut_group, Bacteroides RF16 group* and *Prevotella*. Compared with Mongolian cattle, the Bactrian camel microbial co-occurrence network showed higher edge number, graph density and average degree, but lower network diameter, modularity and clustering coefficient.

## 4 Discussion

Gastrointestinal homeostasis in ruminants depends mainly on coordinated interactions between microbial communities and the host. Microorganisms partially convert nitrogen-containing compounds, such as dietary proteins, into microbial proteins that provide amino acids to the host, and metabolize carbohydrates, including cellulose, hemicellulose and starch, into volatile fatty acids that provide energy (Power et al., 2017). In turn, the host provides a suitable living environment for gut microorganisms, including stable temperature, pH, adequate nutrients and appropriate oxygen conditions, thereby jointly maintaining intestinal microecological stability and function. Ruminant gut microbiota can be acquired from the environment from birth, and its structure and composition largely depend on age, diet, lifestyle, environmental hygiene and disease status (Guo et al., 2020). In the desert grassland ecosystem of the Alxa region, Bactrian camels and Mongolian cattle graze naturally throughout the year and are less affected by external management. Their gut microbial communities and composition therefore remain relatively close to a natural state. Accordingly, Mongolian cattle and Bactrian camels have evolved distinctive digestive systems and dietary habits to adapt to harsh living environments.

In this study, the Shannon index was significantly higher and the Simpson index was significantly lower in Bactrian camel samples than in Mongolian cattle samples (*P* < 0.05), indicating significantly greater microbial community diversity in Bactrian camels. Higher microbial diversity can improve the fermentation efficiency of forage fiber (Liu et al., 2020) and enhance the stability of the host microbial ecosystem, suggesting that Bactrian camels have stronger ecological adaptability and stability than Mongolian cattle (Spor et al., 2011). Bactrian camels feed extensively and directly consume salt-rich xerophytic shrubs and coarse plants close to the ground, and they show greater tolerance to antinutritional factors such as tannins (Gharechahi et al., 2015). By contrast, Mongolian cattle show stronger dietary selectivity and prefer herbaceous grasses, which may help maintain a relatively stable microbial community structure. As a result, their adaptability to extreme environmental fluctuations may be weaker than that of Bactrian camels. Environmental microorganisms are a major source of host-associated microbes, and differences in their composition and intake, together with internal host genetic selection, may ultimately be reflected as observable differences in microbial community structure (Dapa et al., 2023). A more diverse microbial community provides the host with stronger digestive and metabolic capacity, enabling efficient degradation of different plant types and supporting survival and health during periods of nutritional scarcity (Heiman and Greenway, 2016).

PCoA and NMDS analyses showed that fecal samples from Bactrian camels and Mongolian cattle were significantly separated, indicating substantial differences in microbial community composition between the two groups. These differences are mainly associated with host genetic background and digestive physiological structure (Bubier et al., 2021). The rumen of the Bactrian camel has specialized glandular areas and well-developed mucosal folds, which can secrete mucus and urea. It participates in nitrogen cycling and water-salt metabolism regulation. At the same time, its reticulum structure is simplified and the rate of food bolus passage is faster. These anatomical and physiological differences result in different colonization selection pressures on intestinal microorganisms for the two species, thereby forming their respective unique core microbiota structures. It is worth noting that in the PCoA diagram, the samples of the dromedary camels show a wider dispersion in the PC2 direction compared to the Mongolian cattle, indicating that the microbial community variability among dromedary camels is more prominent. This high heterogeneity among individuals may stem from the following factors: firstly, the adaptation strategy of Bactrian camels to the arid desert environment has greater individual plasticity. When their intestinal microbiota respond to fluctuating water and nutrient supply, different individuals may rely on different combinations of microbiota with higher functional redundancy to maintain digestive homeostasis; Secondly, Bactrian camels possess a unique immune-microbial interaction regulatory mechanism. The differentiated mucosal immune responses of individuals may have a differentiated screening effect on the colonization of specific microorganisms. Ming et al. (2017) collected fecal samples from Inner Mongolian cattle, domestic Bactrian camels in Inner Mongolia, domestic Bactrian camels in Mongolia and wild Bactrian camels in Mongolia. Based on 16S rRNA gene sequencing, they found significant microbial community differences between Inner Mongolian cattle and the three Bactrian camel populations, and identified phyla in Inner Mongolian cattle and domestic Bactrian camels from Inner Mongolia that were absent from wild Bactrian camels in Mongolia. Bi et al. (2019) compared the effects of different feeding modes, including maternal nursing and artificial feeding, on early gut microbial composition in newborn goats and found that artificial feeding significantly increased bacterial abundance in the initial gut microbiota of newborn kids. Shah et al. (2024) compared gut microbial diversity between wild and domestic yaks on the Qinghai-Tibetan Plateau and found that wild yaks had higher microbial diversity than domestic yaks, with greater relative abundance of *Bacillota* and stronger fiber-degrading capacity. Liu et al. (2022) found that, because of harsh environmental conditions and nutritional deficiency on the Qinghai-Tibetan Plateau, yak diarrhea occurs frequently; compared with healthy yaks, diarrheic yaks showed markedly reduced alpha diversity in intestinal bacterial and fungal communities, and several bacterial genera were not detected. It indicates that environmental stress and changes in health status can also intensify the differentiation of microbiota among individuals within a population. The intestinal microbiota of camels has a high degree of individual diversity. Its formation is influenced by individual differences such as the host's genetic background, unique digestive physiological characteristics, and the plasticity of individual adaptation strategies to the environment. At the same time, it is also affected by the combined effects of long-term natural grazing conditions and nutritional status. These factors superimpose on each other, generating complex effects. The significant degree of dispersion along the PC2 axis in the PCoA diagram visually reflects the result of the combined action of multiple factors.

At the phylum level, 10 phyla were identified in fecal samples from both Mongolian cattle and Bactrian camels, with *Bacillota* and Bacteroidota being the dominant and most abundant phyla. The relative abundances of the subdominant phyla *Pseudomonadota, Spirochaetota* and *Verrucomicrobiota* were significantly higher in Bactrian camel fecal microbiota than in Mongolian cattle. *Bacillota* is mainly associated with carbohydrate and protein absorption, whereas *Bacteroidota* can degrade dietary fiber that cannot be directly digested by the host, as well as mucin polysaccharides in intestinal mucus. By encoding large numbers of carbohydrate-active enzymes, *Bacteroidota* can ferment polysaccharides into short-chain fatty acids, providing additional energy to the host and helping maintain gut health (Thomas et al., 2011). Intestinal *Pseudomonadota* abundance is a key marker for evaluating mammalian gut health, with elevated levels typically indicating intestinal *dysbiosis* (Shin et al., 2015). In the present study, the higher relative abundance of *Pseudomonadota* may be related to the unique physiological structure and feeding habits of Bactrian camels. *Spirochaetota* has strong polysaccharide fermentation capacity and may enhance the ability of Bactrian camels to degrade high-fiber desert plants rich in secondary metabolites, such as tannins. *Verrucomicrobiota* can improve host metabolism and maintain intestinal mucosal immune homeostasis, and it contains a broad range of glycoside hydrolases (Martinez-Garcia et al., 2012). These findings suggest that, compared with Mongolian cattle, Bactrian camels may have stronger immune capacity and greater adaptation to the local environment.

Bactrian camels and Mongolian cattle share core dominant genera such as *UCG-005, Rikenellaceae_RC9_gut_group, UCG-010* and *Bacteroides* at the genus level, but there are significant differences in the relative abundance and community composition of each genus. LEfSe analysis further showed that bacteria related to cellulose degradation were differentially enriched between the two species, such as *Bacillus* and *Ruminococcus* in Mongolian cattle. The microbial community of Bactrian camel was significantly changed in *Spirochaetota, Pseudomonadota, Treponema, Christenseniaceae _R_7group* and *Succinivibrio*. Rabee et al. (2021) found that during the degradation of barley straw by camels and sheep, Ruminococcus, RC9, and yeast showed higher activity. Subsequent studies have confirmed that the type of forage can significantly regulate the abundance of *Ruminococcus, Prevotella, Butyrivibrio* and *Rikenellaceae_RC9_gut_group* in Bactrian camels (Rabee, 2022). The enrichment characteristics of the above-mentioned bacterial genera in Bactrian camels and Mongolian cattle are highly consistent with their respective feeding strategies and the ability to utilize low-quality roughage, indicating that the differences in host feeding habits are the key external factors shaping the differentiation of intestinal microbiota. *Rikenellaceae_RC9_gut_group* is a key fiber-degrading and acetate-producing bacterium in the rumen and hindgut microbiome of ruminants and provides energy to the host (Xue et al., 2025). *Ruminococcus* is a dominant fiber-degrading bacterium in the gastrointestinal mucosa of herbivores and can produce cellulases involved in fiber fermentation and volatile fatty acid metabolism (Li et al., 2019). *Bacteroides* and *Treponema* also have strong polysaccharide-degrading capacity and produce volatile fatty acids (Hinsu et al., 2021; Difford et al., 2018). The enrichment of differences in these functional flora between the two species provides a potential microbiological explanation for their survival and energy metabolism under low-quality roughage conditions. The rumen, as the core fermentation chamber of ruminants, has a unique anaerobic environment, solid-liquid two-phase retention and selective retention mechanism, which determines that there are essential differences between the anterior stomach and hindgut in terms of fiber degradation efficiency, volatile fatty acid production spectrum and microbial interaction network (Mizrahi et al., 2021). He et al. (2018) found by comparing the microbial flora in different gastrointestinal parts of Bactrian camels that the abundance of unclassified *Ruminococcus* and *Ackermansia* was relatively high in the large intestine, while *Clostridium* and *Bacteroides* were relatively enriched in the forestomach and small intestine. Cheng et al. (2024) found that *Prevotella, Fibrobacter* and *Succinivibrio* had higher relative abundances in the duodenum. While identifying these differences, this study also detected a shared core microbiota composed mainly of *Bacillota* and *Bacteroidota*, consistent with the findings of Rabee et al. (2022). This result indicates that, despite host differences, the basic fermentation functions performed by microbial communities are relatively conserved under similar dietary backgrounds.

Under the same living environment, the gut microbial co-occurrence networks of Bactrian camels and Mongolian cattle showed markedly different topological characteristics. The Bactrian camel co-occurrence network had higher edge number, graph density and average degree, together with a lower network diameter, indicating a more closely connected pattern of microbial interactions in the camel gut. Such a compact network structure and microbial complexity are sufficient to support an efficient and complex metabolic environment centered on *UCG-005, Rikenellaceae_RC9_gut_group* and *Succinivibrio*. This may strengthen fiber degradation mediated by these genera and facilitate efficient food degradation and energy acquisition by Bactrian camels under desert forage conditions characterized by high fiber, low water availability and poor nutrient supply (Lavrentyeva et al., 2024). By contrast, the Mongolian cattle network showed higher modularity and network diameter, with core taxa including *Rikenellaceae_RC9_gut_group, Bacteroides RF16 group, Prevotella, unclassified Lachnospiraceae* and *Roseburia*. This indicates a clearer functional modular division in the cattle gut microbiota, which may be related to the forage environment and host immune regulation in Mongolian cattle (Jiang et al., 2025).

## 5 Conclusions

In summary, this study used high-throughput 16S rRNA gene sequencing to analyze differences in fecal microbial community composition and diversity between Bactrian camels and Mongolian cattle. From a microbial perspective, it preliminarily revealed that in the same environment, the intestinal microbial community of Bactrian camels has a greater species diversity compared to that of Mongolian cattle. The two species are also shaping their own intestinal microbial communities differently to maintain their own survival.

## Data Availability

The 16S rRNA data that support the findings of this study are available from NCBI. The BioProject number is PRJNA1473640.
